# Ischemic Stroke Induces Skeletal Muscle Damage and Alters Transcriptome Profile in Rats

**DOI:** 10.3390/jcm12020547

**Published:** 2023-01-09

**Authors:** Hu Qi, Xiangyu Li, Xiumeng Zhang, Bin Li, Dan Tian, Dejian Wang, Ruocong Yang, Nan Zeng

**Affiliations:** 1 State Key Laboratory of Southwestern Chinese Medicine Resources, Chengdu University of Traditional Chinese Medicine, Chengdu 611137, China; 2 School of Pharmacy, Chengdu University of Traditional Chinese Medicine, Chengdu 611137, China; 3 Acupuncture and Tuina School, Chengdu University of Traditional Chinese Medicine, Chengdu 611137, China

**Keywords:** ischemic stroke, MCAO, pathological feature, autophagy, mitochondri

## Abstract

To establish pathological features of skeletal muscle post-stroke and to provide a background for promising interventions. Adult male SD rats were selected and randomly divided into a control group, a sham group, and a middle cerebral artery occlusion (MCAO) group. The tolerance and capability of exercise were separately collected on days 1, 3, 5, and 7 after the MCAO operation. The neurological deficits, brain infarct volume, soleus histopathology, mRNA-seq analysis, flow cytometry, immunofluorescence, and protein expression analysis were performed on the seventh day. Rats in the MCAO group showed that soleus tissue weight, pulling force, exercise capacity, endurance, and muscle structure were significantly decreased. Moreover, the RNA sequencing array revealed that mitochondrial-mediated autophagy was the critical pathological process, and the result of transcriptomic findings was confirmed at the translational level. The mitochondrial membrane potential and the mfn2 and p62 protein expression were decreased, and the Beclin-1, ATG5, Parkin, PINK1, LC3B, and Drp1 expression were upregulated; these results were consistent with immunohistochemistry. This is the first report on the pathological features of limbic symptoms on day 7 after MCAO surgery in rats. In addition, we further confirmed that autophagy is one of the main causative mechanisms of reduced muscle function after stroke.

## 1. Introduction

With a global prevalence of over 10 million in the last years [1], ischaemic stroke is one of the most prevalent diseases worldwide. Besides causing around 5.2% of deaths [2], the survivors experience disability caused by secondary damage after stroke. This damage directly leads to a heavy financial burden on the community and triggers mental illness in stroke patients [3,4]. Although the focal area of stroke is in the central nervous system, growing data suggest that a systematic overview should be used to investigate the pathological process of this disease [5,6,7,8]. Following the onset of stroke, the lesion area located in the infarcted area first leads to the production of local inflammation [6,8]. Activating microglia causes a cascade response of inflammation in the central system to inflict damage [9,10]. Besides the injury in the central system, secondary impairment of peripheral organs or tissues also occur. Due to the disruption of the integrity of the blood–brain barrier, large amounts of central-derived cytocines enter the circulation system and trigger the damage process in peripheral organs [11,12,13,14]. An increasing number of researchers argue that stroke should be studied systematically and comprehensively to improve patients’ quality of life after stroke prognosis [15,16]. Therefore, the knowledge of the pathological process of the post-stroke limb is essential.

The pathological features after post-stroke have been inadequately reported. Although clinical studies suggest that stroke can secondarily induce damage to the heart [17,18,19], facial muscles [20], and skeletal muscles, the pathological features of these peripheral organs, which can be further used for therapeutic research, remains poorly characterized. As a critical basis for knowledge of the disease, animal disease models can demonstrate pathophysiological processes similar to human disease. Being one of the commonly used model animals, the pathophysiological characteristics of rats are similar to those of humans during the stroke process [21]. Moreover, although these morphological changes in muscle were observed in the middle cerebral artery occlusion (MCAO, Table 1) model on rats [22], the detailed pathological features have not been well characterized. Thus, the present study demonstrated the pathological changes and lesion characteristics appearing in the limbs of MCAO model rats with behavioral, electrophysiological, and transcriptomic methods. These findings will provide a pathological background for subsequent studies.

## 2. Materials and Methods

### 2.1. Animals

Male Sprague–Dawley (SD) rats (SPF level, 6–8 weeks old, 220–250 g) were obtained from Beijing Sibei Fu Laboratory Animal Technology Co., Ltd. (Beijing, China) a certificate of conformity no. No110324210107208383. The animal production license is No. Scxk (Beijing, China) 2019-0010. All animals were fed and watered ad libitum for three days, in an environment with a constant temperature of (22–25 °C, 40–60% humidity), light and dark in the environment of 12 h each feeding. This protocol was approved by the Laboratory Animal Ethics Committee of Chengdu University of Traditional Chinese Medicine (No. 20210320).

### 2.2. MCAO Model in Rats

The experimental animals were adaptively kept for 1 week. The weight of the animals was raised to leave the group, and 48 rats conforming to the weight of 240–260 g were selected and randomly divided into control group, model group, and sham group. The middle cerebral artery occlusion (MCAO) modeling was performed as previous literature described [23]. The rats were anesthetized with Tiletamine Hydrochloride and Zolazepam Hydrochloride for injection (Zoletil 50, BN8LXLA) at a dose of 0.1 mL/100 g. After the rats were anesthetized, they were immobilized in the supine position. A 2.5-cm to 4-cm incision was made with surgical scissors between each rat’s larynx and sternum along the tight mid-abdominal line [21]. The left common carotid artery (CCA), external carotid artery (ECA), internal carotid artery (ICA), artery ECA, and internal carotid artery (ICA) were sequentially exposed. A nylon suture with a rounded tip was then gently advanced from the CCA into the lumen of the ICA to close the carotid artery. The sham group was bluntly separated from the tissue with the same operation but without the MCAO operation.

#### 2.2.1. Neurological Deficit Tests

The same observers, blinded to the group assignment, scored neurological deficits for animals at the time of 24 h after surgery [24]. The Longa test was used for this evaluation for five grades: (1) grade 0, symptoms without neurological impairment; (2) grade 1, failure to extend left forepaw fully when lifting the rats’ tail; (3) grade 2, circling to the left side while walking; (4) grade 3, walking hard and circling to the left; and (5) grade 4, no spontaneous walking and being depressed.

#### 2.2.2. Tolerance and Capability of Exercise

Animal weights were separately collected on days 1, 3, 5, and 7 after the MCAO operation. Rats were on the animal treadmill (SANS, SA101B) to test their tolerance and exercise capability [25]. The instrument was operated according to the operation manual. The parameters were set as follows: Ambient temperature was maintained between 24–28 °C. The exercise time was set as 5 min. The stimulation current was 1.50 mA, and the speed was 10 m/min. Six test lanes were run simultaneously. Each group had two animals participating in the test in parallel.

Grasping force was tested by the SA417 grasping force tester model (Jiangsu Science Biotechnology Co., Ltd., Suzhou, China), which was used to evaluate the forelimb tension of the subject animal [26]. The operation was carried out by following the operation manual. Briefly, the animal was first placed horizontally in the prone position on the grasping board. When the animal’s forelimbs held the gripping plate, the tail of the animal was lifted, and the animal’s hindlimbs were suspended in the air and pulled backwards at a constant horizontal speed. The data were recorded when the animal’s forelimb was released from the gripping plate.

#### 2.2.3. Muscle Electrical Signal Acquisition

Soleus muscles were used to collect the muscle’s electrical information. After the surface hair was removed, the electrodes were inserted into the soleus muscle at an angle of 45° [27]. The muscle electrical signals were acquired through the RM6240XC multi-channel physiological signal acquisition and processing system (Chengdu Instrument Factory) with a stimulation intensity of 1.5 V. After the acquisition, the electrode insertion site was disinfected with iodine.

#### 2.2.4. Evaluation of Cerebral Infarct Size

The rats were anesthetized and placed supine on the operating table. After exposing the heart, cardiac perfusion was performed with saline. When the blood flow was clarified, the skull was carefully opened, and the brain was removed [28]. TTC staining experiments were performed as described previously [29]. Staining results were captured and analyzed with ImageJ software (Version 1.53v, National Institutes of Health, Bethesda, MD, USA).

#### 2.2.5. Length and Weight of Soleus Muscle

The soleus muscles were collected on the seventh day after the MCAO operation. After being anesthetized, the animals were placed on the operating table in a supine position. The hair on the legs is removed, and the skin and surface fascia are bluntly peeled. Then the muscles were carefully stripped to avoid errors caused by manual tugging. For each group of animals, five right hind limb (affected side) soleus muscles were randomly selected for analysis [30].

#### 2.2.6. Hematoxylin and Eosin (H&E) Staining

Statistical analysis of muscle fiber cross-sectional area was performed on sections wanting the same field of view and at the same magnification [31]. The right soleus muscle of rats was quickly removed and placed in 4% para-formaldehyde for 48 h. It was then embedded in paraffin and cut into 4-μm sections. The sections were placed in warm water extension at 42 °C and subsequently dried at 60 °C. After drying and sealing the slices, the staining was performed with hematoxylin and eosin according to the standard protocol [32]. Observations were performed under a 200× bright field.

The ultrastructure of the flounder muscle was observed by transmission electron microscopy. Flounder muscle tissue was fixed in 2.5% frozen glutaraldehyde in 0.1 mol/L PBS and stored at 4 °C. After 24 h, muscle sections were fixed in 1% osmium with nickel oxide for 4 h, dehydrated in graded acetone solution, and impregnated with propylene oxide. The copper mesh was then stained after slicing into 60–90 nm sections using an ultrathin slicer. Transmission electron microscopy (JEOL, JEM-1400FLASH) was used to capture images.

#### 2.2.7. mRNA Sequencing and Analysis Methods

Total RNA was isolated from each soleus muscle sample using the RNA mini kit (Qiagen, Hilden, Germany) [33]. RNA quality was examined using gel electrophoresis and Qubit (Thermo, Waltham, MA, USA). mRNA-seq was performed in the transcriptome and genome analysis laboratory (TAL) of Genergy Biotechnology Co. Ltd. (Shanghai, China) [34]. Library preparation was conducted with instruction of the Quarseq RNA library-Single-s (P9009-A1), using 1 µg of total RNA as the starting material. A fluorescence-based Qubit™ dsDNA HS Assay Kit from Thermo Fisher Scientific (Waltham, MA, USA) was used to accurately quantify cDNA libraries. The size of the final cDNA libraries was determined using the DNA 1000 chip (280 bp) on the Bioanalyzer 2100 (Agilent). The cDNA libraries were amplified for double-ended simultaneous sequencing mode via Illumina PE150 (Illumina, San Diego, CA, USA). Sequence images were transformed into .bcl files with the Base-Caller (Illumina) software. Then they de-multiplexed them into *.fastq* format files with CASAVA (vision 1.8.2). Sequences were aligned to the genome reference sequence of Rattus norvegicus (GRCm38/mm10). The alignment was performed using STAR software (version 2.3.0e), allowing two mismatches within 50 bases. Subsequently, the conversion of resulting SAM files to sorted BAM files, filtering of unique hits, and counting was conducted with SAMtools (version 0.1.19) and HTSeq (version 0.6.1p1). Data were preprocessed and analyzed in the R/Bioconductor environment (www.bioconductor.org, accessed on 11 October 2022) using the DESeq2 package (version 1.8). Specifically, the data were normalized and tested for differentially expressed genes based on a generalized linear model likelihood ratio test, assuming contrary binomial data distribution.

A minimum of |log2 filtered candidate genes (fold change)| > 1, and the corrected P value of a false discovery rate was < 0.05. Gene annotationwas performed using Rattus norvegicus sapiens entries from the DAVID database (https://david.ncifcrf.gov/, accessed on 21 November 2022). TB tools were used to visualize enrichment analysis results.

#### 2.2.8. Mitochondrial Membrane Potential Determination

The primary cells were extracted using the Collagenase II (Yeasen Biotechnology Shanghai Ltd., C7222210, Shanghai, China) kit. The soleus muscle was cut into 3–4 mm pieces with a sterile scalpel and washed several times with HBSS (Yeasen, H7214590) containing Ca^2+^ and Mg^2+^. Subsequently, the collagenase HBSS solution at a concentration of 2 mg/mL was added and incubated at 37 °C for 6 h. After digestion, the cells were washed with HBSS solution and collected with a nylon mesh sieve, adjusting the cell concentration to 1 × 10^6^ cells/mL [35].

For staining analysis, the mitochondrial membrane potential was determined by JC-1 (Beyotime, Jiangsu, China, C2005). Soleus muscle cells were washed with PBS, and 1 mL of RPMI 1640 (Thermo Fisher, 812698) was added. The 1 mL of JC-1 staining working solution was added to the reaction system and thoroughly mixed. The reaction system was incubated for 20 min at 37 °C. After incubation, the cells were centrifuged and resuspended twice using JC-1 staining buffer wash to wash away any residual JC-1 staining solution. After the last centrifugation, resuspension was performed with 2 mL RPMI 1640 and observed in FITC and PE channels by flow cytometry [36].

#### 2.2.9. Immunohistochemical Analysis

The rat soleus muscle was dissected and fixed in a 4% paraformaldehyde solution. After 24 h, the fixed tissues were embedded in paraffin and cut into 4-μm sections. Subsequently, antigen repair was performed at pH = 9.0 in EDTA. Next, after soaking in 3% H_2_O_2_ for 30 min at room temperature, tissue slides were incubated overnight at 4 °C with antibody buffer containing LC3B (Abmart, Berkeley Heights, NJ, USA, T55992S) [37] and p62 (Proteintech, Rosemont, IL, USA, 18420-1-AP) [38] antibodies. After incubation with the primary antibody, the residual antibody was washed away using PBS. Then, goat anti-mouse IgG (Abcam, Cambridge, UK, ab205718) was incubated for 30 min at room temperature and photographed under a bright field. The ratio of positive regions was statistically analyzed using ImageJ.

The soleus muscle was dissected and fixed in 4% paraformaldehyde solution, then embedded in paraffin and cut into 4 μm sections. It was then antigenically repaired with EDTA at pH 9.0, immersed in 3% H_2_O_2_ for 30 min at room temperature, and incubated overnight at 4° with 10% goat serum containing LC3B (Abmart, T55992S) and p62 (proteintech, 184201AP). The next day, the primary antibody was washed, then stained after incubation with Goat Anti-Mouse IgG (Abcam, ab205718) and photographed by white light microscopy (Olympus, Tokyo, Japan, CX31). Statistical analysis of the rate of positive regions was performed using ImageJ.

#### 2.2.10. Western Blot Analysis

Homogenized halibut muscle tissue was lysed in ice-cold radioimmunoprecipitation assay (RIPA) buffer (Beyotime, Shanghai, China), which contained 1% protease and 1% phosphatase inhibitors. Total protein concentration was quantified by using a BCA protein assay kit (Beyotime, Shanghai, China). Equal amounts of total protein in each group were separated electrophoretically using 10% sodium dodecyl sulfate (SDS-PAGE) and subsequently transferred into the polyvinylidene difluoride (PVDF) membranes (Merck Millipore, Darmstadt, Germany) that were activated with anhydrous methanol. Afterward, the membranes were blocked with 5% BSA for 2 h at room temperature and then incubated overnight at 4 °C with the following specific primary antibodies: β-Actin antibody (1:2000, Abmart, T40104F), LC3B Antibody (1:2000, Abmart, T55992S), Mitofusin 2 Antibody (1:2000, Abmart, T56638S), Parkin Antibody (1:2000, Abmart, T56641S), Drp1 Antibody (Abmart, 1:2000, TD7037S), PINK1 (1:2000, Abmart, PA5801S), p62 (1:5000, Proteintech, 18420-1-AP), Beclin-1 (1:2000, Abmart, Q14457), ATG5 (1:2000, Abmart, Q9H1Y0), GAPDH (1:2000, Servicebio, Ghent, Belgium, GB11002).

After washing 5 times with 1× TBST for a total of 30 min, the membranes were incubated for 1.5 h with the appropriate HRPconjugated secondary antibodies at room temperature. The bands were visualized using an enhanced chemiluminescence (ECL) kit (4A Biotech Co., Ltd., Beijing, China). The images of bands were taken using the ChemiDocTM Imaging System (Bio-Rad, Hercules, CA, USA) and adjusted to proper exposure contrast. They were further quantified using Image Lab 3.0 software (Bio-Rad, Hercules, CA, USA) and statistically analyzed using SPSS (IBM Corp., Armonk, NY, USA) and GraphPad 8.0 (GraphPad Software, Inc., La Jolla, CA, USA).

### 2.3. Statistical Analysis

SPSS software was used for statistical analysis of the data, and all results data were expressed as ± s. If the data conformed to a normal distribution, the one-way analysis of variance (One-way ANOVA) was used for variance detection. Moreover, for post hoc comparisons, Tukey’s multiple comparisons were further employed for within-group detection of the chi-square data, while Tambane’s T2 test was performed for group comparisons of data with heterogeneous variances. Once the data failed to conform to the normal distribution, the non-parametric test rank sum test (Mann–Whitney U) was used. *p* < 0.05 was considered statistically significant.

## 3. Results

### 3.1. Reduction in Limb Function after Stroke

**Figure 1:**
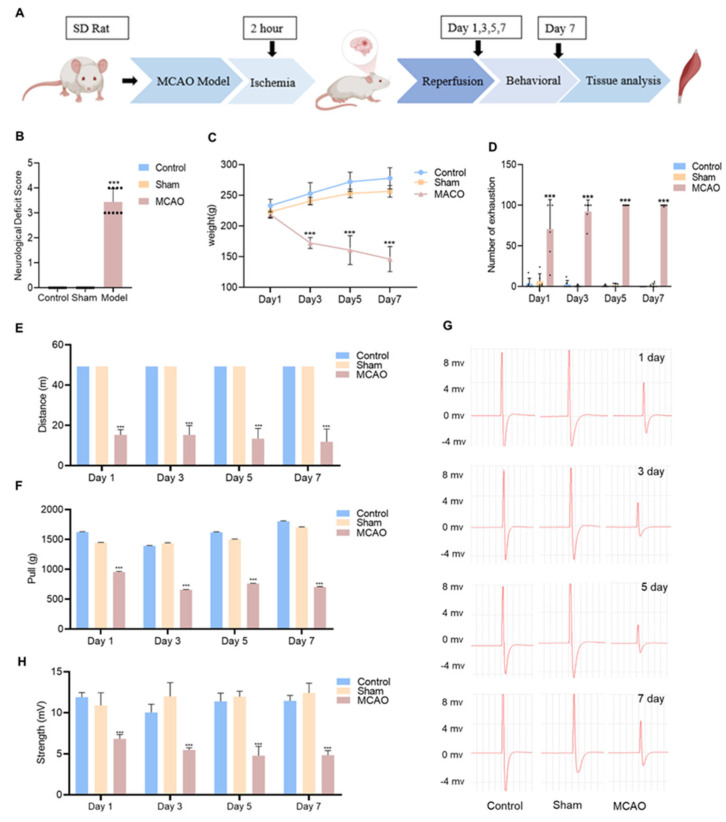
Behavioral changes in MCAO rats. (**A**) Behavioral experimental design for studying changes in muscle function after middle cerebral artery occlusion (MCAO) model rats. (**B**) Assessment of neurological deficits in Zea-longa rats (*n* = 9). (**C**) Body weight change of rats (*n* = 9). (**D**) The number of 5 min of force exhaustion collected by the small animal treadmill (*n* = 9). (**E**) 5 min running distance (*n* = 9). (**F**) Grip strength in rats (*n* = 9). (**G**) Muscle electrical signal intensity (*n* = 8). (**H**) Visual diagram of muscle electrical signal intensity. *** *p* < 0.001 vs. the control group, no significant difference between the control group and sham.

The study schedule is shown in Figure 1A. The Longa score was performed on the first day after the MCAO operation to access the motor changes [24]. The animal score of the MCAO group was (3.4 ± 0.55), with a significant difference (*p* < 0.001) compared to the control and sham group rats (Figure 1B and Supplementary Table S1). The damage was confirmed via TTC staining on the seventh day after the MCAO operation (Supplementary Figures S1 and S2, Supplementary Table S2). Besides, the body weight detection results showed that the MCAO model group’s animals exhibited a significant decrease since the third day after the stroke occur (Figure 1C and Supplementary Table S3).

Different behavioral changes, including motor function, motility, motor endurance, and tensile strength, were detected to reflect the changes by stroke (Figure 1D–G and Supplementary Tables S4–S7). Treadmill experiments were used to evaluate motor endurance and motility. There were no significant differences in the number of exhaustion and movement distances between the control and sham groups. In comparison with the sham-operated group, the animals of the MCAO group showed a significant (*p* < 0.001) increase in the number of exhaustions at all time points, but stabilized at a high level on the third day (Figure 1D, Supplementary Table S4). As for the motor ability reflected by moving distances, the results of the MCAO group showed a significant (*p* < 0.001) decrease from the onset of stroke (Figure 1E, Supplementary Table S5). Muscle strength was examined with a tensioner. The results showed (Figure 1F and Supplementary Table S6) that the animals in the MCAO group showed significant alterations from the first day after stroke onset compared with the sham-operated group. On the third day after the MCAO operation, the muscle strength was consistently reduced and remained stable at follow-up. Muscle electrical signal monitoring is a vital assessment method for muscle function. After surgery, the intensity of muscle electrical signals in the soleus muscle of rats in the MCAO group significantly decreased (*p* < 0.001) and exhibited a continuous decrease with the progression of stroke.

### 3.2. The Pathological Damage on Skeletal Muscle

**Figure 2:**
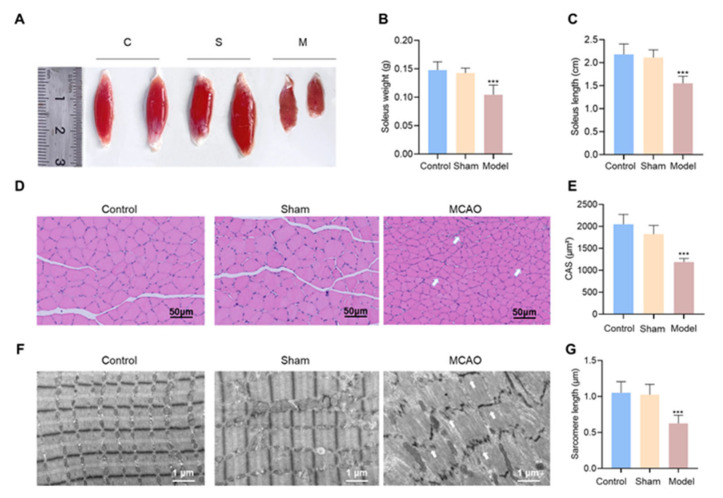
Pathobiological changes in the right hind limb soleus muscle on the affected side of MCAO rats. (**A**) Representative images of the soleus muscle of affected lower limb of rats. (**B**) Weight of affected soleus muscle. (**C**) Length of the affected soleus muscle. (**D**) Representative pictures and analysis of H&E staining of the rat soleus muscle (*n* = 6). (**E**) Cross-sectional area of muscle fibers in the same area of the rat soleus muscle, same field of view (200×). (**F**) The representative images for TEM detection of cardiac ultrastructure were shown. (**G**) Analysis of soleus sarcomere length. C, control, S, sham, M, MACO, CAS, Cross-sectional area, *** *p* < 0.001 vs. the control group. H&E, hematoxylin, and eosin, TEM, transmission electron microscopy.

Since the soleus muscle is a representative motor muscle among the skeletal muscles, we used it as an illustration for the test. The morphological characteristics of the soleus muscle displayed that the MCAO operation resulted in a reduction (*p* < 0.001) in both the weight and length of the soleus muscle as compared to the sham group (Figure 2A–C, Supplementary Table S8). Morphological differences in tissue-level appearance suggest that lesions due to MCAO may be associated with structural injury of muscle cells.

To further investigate the damage of soleus muscles in MCAO model rats, we performed H&E staining and transmission electron microscopy (TEM) approaches. As shown in Figure 2D, muscle cells in the control and sham groups had normal-sized cytosomes with clear borders and regular arrangement. In contrast, the muscle cells in the MCAO group were significantly diminished and the cross-sectional area was notably reduced (*p* < 0.001). In more detail, ultrastructure displayed that muscle cells in the control and sham groups had normal intracellular morphological structures; dark bands were intact in the structures, and the Z and M lines were flat and complete. Comparatively, fundamental myocytes in the MCAO group disappeared and intact myofibrils could not be identified. The Z line was distorted, the M line was blurred, and myocytes had mildly cleaved myogenic fibers. This suggests that there was substantial damage to muscle cell integrity (Figure 2F). Furthermore, the cross-sectional area (CSA) of muscles in H&E staining and the length of muscle segments in ultrastructure were counted, and significant reduction was found in the MCAO model group in comparison to the control and sham groups (Figure 2E,G, Supplementary Tables S9 and S10). This suggests that the decrease in motor function after ischemic stroke might be associated with alterations in muscle structure.

### 3.3. Alterations of the Transcriptome in Skeletal Muscle after Stroke

**Figure 3:**
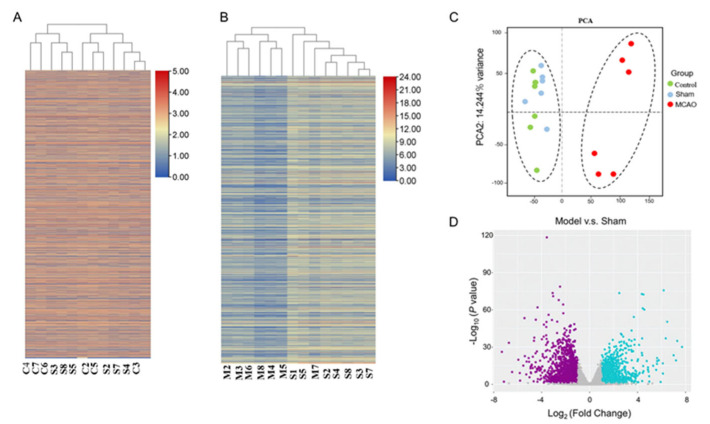
mRNA−seq: differential gene expression analysis (**A**,**B**) Hierarchical cluster analysis of the significantly upregulated and downregulated mRNAs, each column represented a sample, and each row represented an mRNA. (**C**) PCA analysis of control, sham, and MCAO. (**D**) Volcano plot of differentially expressed genes (DEGs). Blue represents up-regulated genes, Purple represents down-regulated genes, and gray dots represent nonsignificantly DEGs.

We next carried out RNA sequence analysis to reveal the molecular mechanism of muscle injury after ischemic stroke. As shown in Figure 3A, no difference was observed in expression between the control and sham groups. This suggests no substantial muscle damage from surgical operations other than middle cerebral artery occlusion. In contrast, the clustering results of the MCAO model and the sham-operated group suggest that the pathological process of ischemic stroke materially affects the pathophysiologic states of skeletal muscle (Figure 3B). The further principal component analysis (PCA) in Figure 3C demonstrated that the RNA expression data results could clearly distinguish the animals in all groups into the MCAO model-affected group and the group without MCAO intervention. The PCA result suggests that it is reasonable to use middle cerebral artery occlusion as the unique variable for follow-up analysis by using the sham-operated and MCAO model groups. With a threshold screen of fold change values greater than 2 and false discovery rate (FDR) values less than 0.05, 888 genes were significantly up-regulated in the MCAO model group compared to the sham-operated group, while 1017 genes were significantly down-regulated.

**Figure 4:**
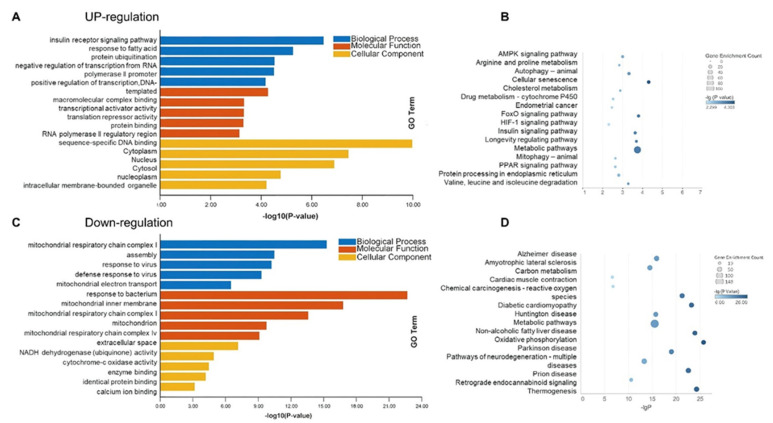
Enrichment of differential expression genes between sham group and MCAO group. (**A**,**B**) the up−regulation genes of GO and KEGG analysis. (**C**,**D**) the down−regulation genes of GO and KEGG analysis.

In addition, 1905 differential genes in the sham and MCAO groups were employed for enrichment analysis to investigate the pathological processes in skeletal muscle after ischemic stroke. As shown in Figure 4A,B, the enrichment analysis of the 888 genes significantly upregulated revealed remarkable changes in intracellular transcriptional levels (negative regulation of transcription from RNA polymerase II promoter, positive regulation of transcription, DNA-templated; FoxO signaling pathway), which may occur in association with the onset of autophagic processes (Autophagy-animal, Mitophagy-animal). Besides, enrichment analysis of 1017 significantly down-regulated genes in Figure 4C,D implied that the lesion site of skeletal muscle damage after stroke was primarily the mitochondria (mitochondrial inner membrane; mitochondrial respiratory chain complex I; mitochondrion; mitochondrial respiratory chain complex IV) and that the pathological process may be associated with a decline in mitochondrial function (mitochondrial respiratory chain complex I assembly; mitochondrial electron transport, NADH to ubiquinone; NADH dehydrogenase (ubiquinone) activity; cytochrome-c oxidase activity).

### 3.4. Mitochondrial Dysfunction

**Figure 5:**
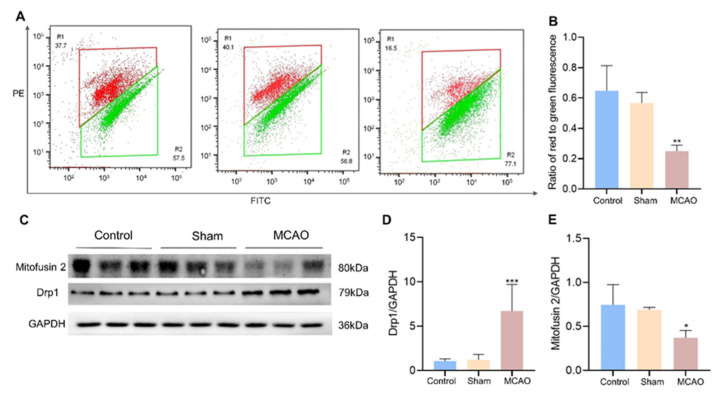
Effect of MCAO on mitochondrial functional impairment in rat soleus muscle. (**A**) Representative pictures and analysis of mitochondrial membrane potential changes detected by flow cytometry (*n* = 4–6). (**B**) The ratio of red fluorescence to green fluorescence. (**C**) Western blot showing the protein expression levels of Mitofusin 2, DRP1, and GAPDH in soleus muscle tissue. (**D**,**E**) Relative protein expression for DRP1, Mitofusin 2, and GAPDH were quantified by densitometry based on immunoblot images (*n* = 6–10). Results are presented as mean ± SD. * *p* < 0.05, ** *p* < 0.01, *** *p* < 0.001 vs. the control group and sham group.

Prompted by the transcriptomic results, we further detected the mitochondrial status. We measured the mitochondrial membrane potential by using flow cytometry with JC-1 dye. When the mitochondrial membrane potential is high, JC-1 aggregates in the mitochondrial matrix and forms a polymer, which can produce red fluorescence. In contrast, when the potential difference of mitochondrial membrane is narrow, JC-1 cannot aggregate in the matrix of mitochondria, at which time, JC-1 is monomeric and produces green fluorescence. The results showed that the control and sham groups showed high red fluorescence red and weak green fluorescence (Figure 5A). This indicates that the mitochondrial potential in the control and sham groups was stable and did not produce damage. Comparatively, the green fluorescence of mitochondria was significantly increased (*p* < 0.01) and the red fluorescence was significantly decreased in the MCAO group (Figure 5B and Supplementary Table S11). The result (not shown) was consistent with the effect of the positive reagent Carbonyl cyanide 3-chlorophenylhydrazone (CCCP), suggesting that mitochondria underwent depolarization and decreased mitochondrial membrane potential.

Moreover, the Drp1 protein expression was significantly increased (*p* < 0.001) and the Mfn2 protein was significantly decreased (*p* < 0.05) in the MCAO group (Figure 5C–E and Supplementary Table S12). This suggests that the process of mitochondrial fusion and division in skeletal muscle is remarkably altered after stroke onset.

### 3.5. Stroke-Induced Autophagic Processes in Soleus Muscle

**Figure 6:**
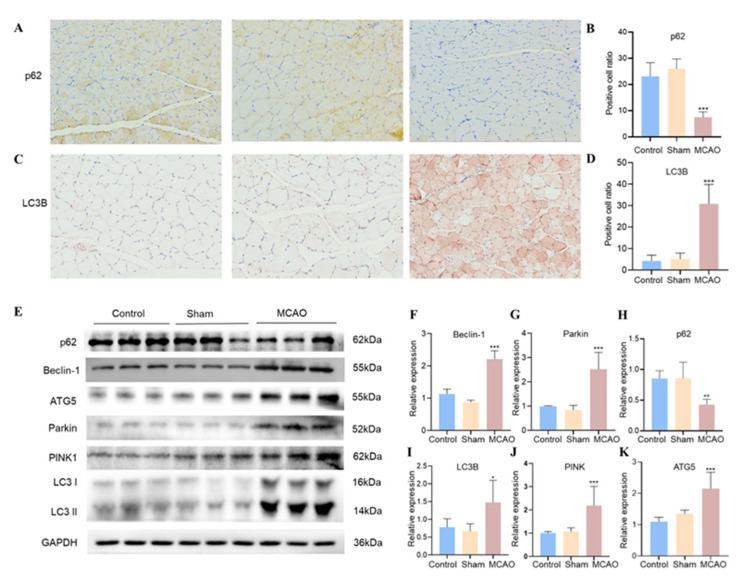
Expression of autophagic proteins in soleus muscle. (**A**) Immunohistochemical analysis of paraffin-embedded soleus tissue slide using p62 antibody. (**B**) p62 positive cell ratio. (**C**) Immunohistochemical analysis of paraffin-embedded soleus tissue slide using p62 antibody. (**D**) LC3B positive cell ratio. (**E**) Western blot bands showing the protein expression levels of p62, Beclin-1, ATG5, Parkin, PINK, LC3BI, LC3BII, and GAPDH. (**F**–**K**) Relative protein expression for p62, Beclin-1, Parkin, PINK, LC3BI, LC3BII, and GAPDH was quantified by densitometry based on immunoblot images (*n* = 6). * *p* < 0.05, ** *p* < 0.01, *** *p* < 0.001 vs. There was no significant difference between the control group and the sham group.

Combining the results of transcriptomic and mitochondria-related assays, we hypothesized that mitochondria-related autophagy might be one of the predominant pathological processes in skeletal muscle during this phase. Thus, we examined the autophagic status within the soleus muscle 7 days after the ischemic stroke. Immunohistochemical results showed that p62 expression was significantly decreased. In contrast, LC3B expression was significantly (*p* < 0.001) increased in rat soleus muscle after MCAO operation (Figure 6A–D and Supplementary Table S13). This was consistent with the outcome from the western blot (Figure 6E,H,I and Supplementary Table S12). The expression of critical proteins in the autophagic process was further detected. We observed that Beclin-1, ATG5, Parkin, and PINK1 were significantly up-regulated in the MCAO group (Figure 6E–K and Supplementary Table S14). These results suggest that stroke secondarily causes a pathological process of autophagy in skeletal muscle.

## 4. Discussion

Widespread muscle damage is reported in the stroke process [39]. This suggests that interventions on skeletal muscle might provide novel options for improving post-stroke rehabilitation [40,41]. However, based on physical interventions, the current therapeutic approach fails to provide individual interventions. One of the reasons for the gap is the lack of pathological evidence for the post-stroke limb.

Although muscle damage is already observed in the rat’s MCAO model [42], the systemic evaluating methods and molecular mechanisms remain largely unknown. Thus, in this study, we have described the changes in limb characteristics after stroke on the MCAO model in rats, ranging from visible limb symptoms to microscopic molecular transmission. Specifically, the evaluation of limb symptom alteration was first achieved through behavioral experiments and electrophysiology, which revealed that limb symptoms do change muscle function after stroke. We then explored the direction of pathological progression using a transcriptomic approach, which resulted in identifying mitochondria-mediated autophagy as the critical pathological process. Additionally, we validated the key proteins in the pathological process of mitophagy.

The normal function of the muscles is the basis for maintaining the correct body movement [43]. Both clinical studies and basic research have reported pathological changes in the function of the limbs following stroke onset [44]. These alterations include muscle atrophy [45], loss, and cachexia. Behavioral assessment after stroke onset usually falls into two main categories: neurological symptom scores [46] and symmetrical balance assessment of the limbs [44]. Despite the current assessment indicators being able to reflect behavioral alterations, they do not precisely reflect skeletal muscle changes after stroke. According to published clinical and basic studies, skeletal muscle injury after stroke primarily occurs in the transverse muscle [47]. Therefore, we refer to the studies from transverse muscle atrophy [48], including the treadmill and pull tests [49]. In this study, we found that the pathological alteration of endurance and exercise in animals under the MCAO model was not synchronized. Specifically, the decrement in exercise tolerance remains stable from the fifth day after the onset of stroke, while exercise capacity, as reflected by exercise distance, varies significantly from the first day after the disease onset. The asynchronous reduction in exercise tolerance and capacity suggests that the damage after the onset of stroke might have progressively influenced the micro-morphological structure of the skeletal muscle.

We further performed tissue-level studies using HE staining and transmission electron microscopy to explore this stage-specific pathology’s morphological basis. Our results confirmed that stroke led to atrophy of skeletal muscle in the model animals compared to the control and sham-operated groups. Significant changes in the structure of the muscle segments were observed at the microscopic level. These phenomena are consistent with clinical reports. These phenomena are consistent with clinical reports and indicate that skeletal muscle pathology after stroke primarily involves atrophy.

Nonetheless, the cause of the emergence of such atrophy involving skeletal muscle during the stroke process has yet to be adequately reported, so we proceeded to use a high-throughput screening strategy to establish the pathological background. Transcriptomics is an experimental approach based on the transcriptional level that can sensitively represent the response of a tissue or cell to external stimuli. Our results demonstrate significant shifts in skeletal muscle transcript levels after stroke. In detail, the critical nodes of autophagy, especially those associated with mitophagy, were significantly upregulated. Simultaneously, mitochondrial function-related pathways were markedly down-regulated. These transcriptional results indicate that the secondary skeletal muscle impairment after stroke correlates with excessive autophagy induced by mitochondrial dysfunction. Autophagy is a process that has both protective and damaging properties. Moderate autophagy helps remove misfolded proteins, toxic substances, and damaged organelles from the cell. However, excessive autophagy can disrupt the enzyme system, causing cellular and tissue damage. Impaired mitochondria are the vital pro-autophagic organelles in the autophagic process. Different degrees of impairment are used to classify mitochondrial damage into functional and morphological injuries. Thus, we further examined the damaged state of skeletal muscle mitochondria on the seventh day after stroke onset.

The decrease in mitochondrial membrane potential occurs before the autophagic process. Mitochondria with reduced membrane potential are preferentially isolated by LC3B-positive structures, irreversibly triggering the mitochondria and preventing the mitochondrial division and fusion process. In turn, the occurrence of mitophagy is promoted. In this study, the electron microscopic results show that, consistent with transcriptomic findings, the morphological features of mitochondria do not change at this stage, 7 days after stroke; meanwhile, Mitofusion 2 and Drp1, proteins that maintain the structural stability of mitochondria, displayed significant differences in expression. These results suggest that functional mitochondrial pathological changes have occurred in skeletal muscle at the time point of 7 days after stroke. However, widespread mitochondrial structural damage has not yet occured. In addition, our results verify the occurrence of autophagy and that one of the lesion locations are the mitochondria.

## 5. Conclusions

To the best of our knowledge, this is the first report on the pathological features of limbic symptoms on day 7 after MCAO surgery in rats. In addition, we further confirmed that autophagy is one of the main causative mechanisms of reduced muscle function after stroke. These findings will potentially provide a pathological background to optimize future rehabilitation treatment.

## Figures and Tables

**Table 1:** List of abbreviations.

Abbreviated Name	Full Name
MCAO	Middle cerebral artery occlusion
SD	Sprague Dawley rats
CCA	Common carotid artery
ECA	External carotid artery
ICA	Internal carotid artery
PBS	Phosphate Buffered Saline
HBSS	Hank’s Balanced Salt Solution
JC-1	Mitochondrial membrane potential assay kit with JC-1
PVDF	Polyvinylidene fluoride
ECL	Enhanced chemiluminescence
TTC	Triphenyltetrazolium chloride
TEM	Transmission electron microscopy
H&E	Hematoxylin-eosin staining
Drp1	Dynamin related protein 1
Mfn2	Mitofusin 2
p62	Sequestosome-1
Beclin-1	Myosin-like BCL2 interacting protein
ATG5	Autophagy related 5 homolog
Pink	PTEN induced putative kinase 1
GAPDH	Glyceraldehyde-3-phosphate dehydrogenase

## Data Availability

Data will be made available on request.

## Supplementary Materials

The following supporting information can be downloaded at: https://www.mdpi.com/article/10.3390/jcm12020547/s1, Figure S1: Triphenyltetrazolium chloride (TTC) stain; Figure S2: Infarct volume of ischemic area; Table S1: Triphenyltetrazolium chloride staining (*n* = 5); Table S2: Neurological deflict score (*n* = 9); Table S3: Body weight (*n* = 7–8); Table S4: The counts of exhaustion (*n* = 6); Table S5: Moving distance (*n* = 5–6); Table S6: Pull strength (*n* = 6–9); Table S7: Muscle electrical signal intensity (n=4–5); Table S8: Soleus weight and soleus length (*n* = 5); Table S9: Muscle cross section (*n* = 5–6); Table S10: Sarcomere length (*n* = 44–47); Table S11: JC-1 ratio of red to green fluorescence (*n* = 4–6); Table S12: The protein expression of Drp1 and mitofusin 2 (*n* = 5); Table S13: LC3B positive cell ratio and p62 positive cell ratio (*n* = 5); Table S14: Protein expression related to autophagic process (*n* = 5).
