# Supplementary material for: Ischemic Stroke Induces Skeletal Muscle Damage and Alters Transcriptome Profile in Rats
Source: J Clin Med. 2023 Jan 9;12(2):547. doi: 10.3390/jcm12020547 (PMC9865444; doi:10.3390/jcm12020547)
Supplement: Supplementary file 1 [file jcm-12-00547-s001.zip › jcm-2141366-supplementary.pdf]

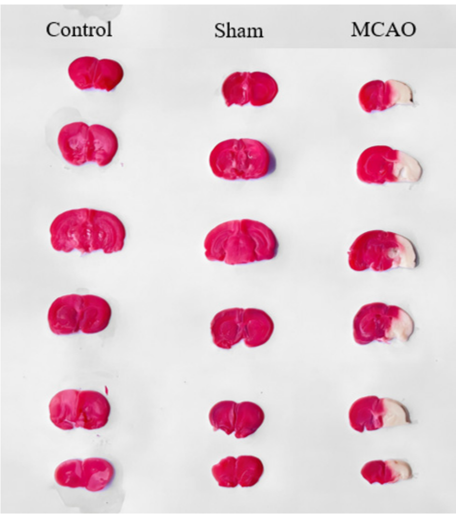


**Supplementary Figure S1.** Triphenyltetrazolium chloride (TTC) stain.

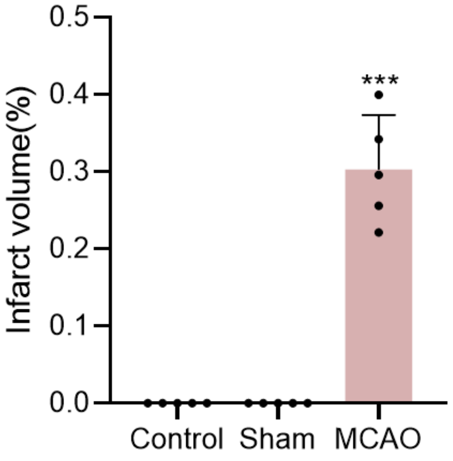


**Supplementary Figure S2.** Infarct volume of ischemic area

**Supplementary Table S1.** Triphenyltetrazolium chloride staining (n=5)

| **Group** | **Infarct volume (%)** | ***P* value** |
|---|---|---|
| Control | 0 | 1.000 |
| Sham | 0 | / |
| MCAO | 0.303±0.070*** | $5.068*10^{-9}$ |

*** $p < 0.001$, vs. the sham group.

**Supplementary Table S2.** Neurological deflict score (n=9)

| **Group** | **Longa score** | ***P* value** |
|---|---|---|
| Control | 0 | 1.000 |
| Sham | 0 | / |
| MCAO | 3.444±0.527*** | 0.000 |

*** $p < 0.001$, vs. the sham group.

**Supplementary Table S3.** Body weight(n=7-8)

| Group | Body weight (g) | | | | | | | |
|---|---|---|---|---|---|---|---|---|
| | **Day 1** | ***P* value** | **Day 3** | ***P* value** | **Day 5** | ***P* value** | **Day 7** | ***P* value** |
| Control | 233.250±10.416 | 0.298 | 252.875±17.931 | 0.055 | 272.000±15.519 | 0.031 | 277.875±17.274 | 0.150 |
| Sham | 223.125±9.046 | / | 240.625±6.070 | / | 253.125±6.875 | / | 256.500±9.426 | / |
| MCAO | 218.500±5.904$^{*}$ | $2.667*10^{-4}$ | 172.375±8.928$^{***}$ | $2.203*10^{-11}$ | 160.714±23.478$^{***}$ | $6.804*10^{-11}$ | 146.142±20.359$^{***}$ | $2.390*10^{-12}$ |

* $p < 0.05$, *** $p < 0.001$, vs. the sham group.

**Supplementary Table S4.** The counts of exhaustion (n=6)

| Group | Exhaution times | | | | | | | |
|---|---|---|---|---|---|---|---|---|
| | **Day 1** | ***P* value** | **Day 3** | ***P* value** | **Day 5** | ***P* value** | **Day 7** | ***P* value** |
| Control | 20.00±39.74 | 0.475 | 2.67±4.84 | 0.670 | 0 | $1.7*10^{-3}$ | 0 | 0.138 |
| Sham | 6.67±9.20 | / | 0.50±1.23 | / | 1.83±2.04 | / | 1.33±2.42 | / |
| MCAO | 70.67±36.25$^{*}$ | $3.11*10^{-4}$ | 92.33±14.00$^{***}$ | $1.03*10^{-12}$ | 100±0.00$^{***}$ | 0.000 | 99.67±0.816$^{***}$ | 0.000 |

* $p < 0.05$, *** $p < 0.001$, vs. the sham group.

**Supplementary Table S5.** Moving distance (n=5-6)

| Group | Distance (m) | | | | | | | |
|---|---|---|---|---|---|---|---|---|
| | **Day 1** | ***P* value** | **Day 3** | ***P* value** | **Day 5** | ***P* value** | **Day 7** | ***P* value** |
| Control | 49.30±0.00 | 1.00 | 49.30±0.00 | 0.224 | 49.30±0.00 | 0.361 | 49.30±0.00 | 1.00 |
| Sham | 49.30±0.00 | / | 42.22±15.83 | / | 41.78±16.81 | / | 49.30±0.00 | / |
| MCAO | 28.90±18.71$^{***}$ | $8.33*10^{-4}$ | 15.26±4.71$^{***}$ | $4.52*10^{-5}$ | 20.60±16.64$^{***}$ | $2.41*10^{-3}$ | 26.80±20.93$^{***}$ | $9.06*10^{-4}$ |

*** $p < 0.001$, vs. the sham group.

**Supplementary Table S6.** Pull strength (n=6-9)

| Group | Pull (g) | | | | | | | |
|---|---|---|---|---|---|---|---|---|
| | **Day 1** | ***P* value** | **Day 3** | ***P* value** | **Day 5** | ***P* value** | **Day 7** | ***P* value** |
| Control | 1622.25±69.22 | $2.97*10^{-4}$ | 1391.88±199.86 | 0.520 | 1620.00±111.41 | 0.134 | 1804.13±123.66 | 0.181 |
| Sham | 1444.33±39.05 | / | 1436.25±124.19 | / | 1500.25±181.94 | / | 1703.50±188.09 | / |
| MCAO | 956.00±136.39*** | $7.35*10^{-10}$ | 657.17±63.97*** | $4.75*10^{-7}$ | 760.14±158.489*** | $9.93*10^{-10}$ | 703.00±112.807*** | $5.58*10^{-13}$ |

*** $p < 0.001$, vs. the sham group.

**Supplementary Table S7.** Muscle electrical signal intensity (n=4-5)

| Group | Electrical signal intensity (mV) | | | | | | | |
|---|---|---|---|---|---|---|---|---|
| | **Day 1** | ***P* value** | **Day 3** | ***P* value** | **Day 5** | ***P* value** | **Day 7** | ***P* value** |
| Control | 11.89±0.56 | 0.185 | 10.03±1.01 | $3.45*10^{-3}$ | 11.39±1.01 | 0.392 | 11.44±0.67 | 0.126 |
| Sham | 10.89±1.54 | / | 11.99±1.67 | / | 11.95±0.68 | / | 12.41±1.20 | / |
| MCAO | 6.85±0.51*** | $1.98*10^{-5}$ | 5.49±0.27*** | $1.08*10^{-6}$ | 4.79±1.12*** | $1.25*10^{-8}$ | 4.84±0.56*** | $2.83*10^{-9}$ |

*** $p < 0.001$, vs. the sham group.

**Supplementary Table S8.** Soleus weight and soleus length (n=5)

| **Group** | **Soleus weight (g)** | ***P* value** | **Soleus length (cm)** | ***P* value** |
|---|---|---|---|---|
| Control | 0.148±0.014 | 0.565 | 2.18±0.23 | 0.573 |
| Sham | 0.143±0.008 | / | 2.11±0.17 | / |
| MCAO | 0.105±0.017*** | $8.84*10^{-5}$ | 1.55±0.15*** | $4.28*10^{-5}$ |

*** $p < 0.001$, vs. the sham group.

**Supplementary Table S9.** Muscle cross section (n=5-6).

| **Group** | **Cross section (μm$^2$)** | ***P* value** |
|---|---|---|
| Control | 2049.96±223.99 | $6.08*10^{-2}$ |
| Sham | 1823.69±200.49 | / |
| MCAO | 1187.52±81.85*** | $4.25*10^{-6}$ |

*** $p < 0.001$, vs. the sham group.

**Supplementary Table S10.** Sarcomere length (n=44-47)

| **Group** | **Sarcomere length (μm)** | ***P* value** |
|---|---|---|
| Control | 1.054±0.154 | 0.345 |
| Sham | 1.026±0.143 | / |
| MCAO | 0.626±0.113*** | 0.000 |

*** $p < 0.001$, vs. the sham group.

**Supplementary Table S11.** JC-1 ratio of red to green fluorescence (n=4-6)

| **Group** | **Ratio of red to green (%)** | ***P* value** |
|---|---|---|
| Control | 0.648±0.165 | 0.274 |
| Sham | 0.567±0.069 | / |
| MCAO | 0.249±0.039** | $6.33*10^{-5}$ |

** $p < 0.01$, vs. the sham group.

**Supplementary Table S12. The protein expression of Drp1 and mitofusin 2** (n=5).

| **Group** | **Drp1** | ***P* value** | **Mitofusin 2** | ***P* value** |
|---|---|---|---|---|
| Control | 1.073±0.251 | 0.861 | 0.745±0.231 | 0.648 |
| Sham | 1.200±0.613 | / | 0.689±0.277 | / |
| MCAO | 6.715±2.990*** | $8.01*10^{-7}$ | 0.371±0.084* | 0.034 |

* $p < 0.05$, ** $p < 0.01$, *** $p < 0.001$, vs. the sham group.

**Supplementary Table S13.** LC3B positive cell ratio and p62 positive cell ratio (n=5).

| **Group** | **Positive cell ratio (%)** | ***P* value** | **Positive cell ratio (%)** | ***P* value** |
|---|---|---|---|---|
| Control | 4.291±2.637 | 0.829 | 23.095±5.251 | 0.257 |
| Sham | 5.090±2.855 | / | 26.027±3.714 | / |
| MCAO | 30.798±9.059*** | $1.18*10^{-6}$ | 7.501±2.039*** | $7.05*10^{-7}$ |

*** $p < 0.001$, vs. the sham group.

**Supplementary Table S14.** Protein expression related to autophagic process (n=5).

| Group | Beclin-1 | *P* value | Parkin | *P* value | p62 | *P* value | LC3B | *P* value | PINK1 | *P* value |
|---|---|---|---|---|---|---|---|---|---|---|
| Control | 1.129±0.155 | 0.129 | 0.991±0.023 | 0.525 | 0.851±0.129 | 0.963 | 0.779±0.237 | 0.627 | 1.008±0.064 | 0.785 |
| Sham | 0.872±0.067 | / | 0.837±0.198 | / | 0.857±0.264 | / | 0.665±0.209 | / | 1.074±0.158 | / |
| MCAO | 2.210±0.259*** | $9.49*10^{-5}$ | 2.523±0.689*** | $1.03*10^{-6}$ | 0.426±0.087** | $1.01*10^{-3}$ | 1.476±0.626** | $6.34*10^{-3}$ | 2.191±0.825*** | $1.15*10^{-4}$ |

* $p < 0.05$, ** $p < 0.01$, *** $p < 0.001$, vs. the sham group.
